# Improving delivery of care in rural emergency departments: a qualitative pilot study mobilizing health professionals, decision-makers and citizens in Baie-Saint-Paul and the Magdalen Islands, Québec, Canada

**DOI:** 10.1186/s12913-020-4916-1

**Published:** 2020-01-29

**Authors:** Richard Fleet, Catherine Turgeon-Pelchat, Mélanie Ann Smithman, Hassane Alami, Jean-Paul Fortin, Julien Poitras, Jean Ouellet, Jocelyn Gravel, Marie-Pierre Renaud, Gilles Dupuis, France Légaré

**Affiliations:** 1 Department of Family and Emergency Medicine, Université Laval, 143 Rue Wolfe, Lévis Québec, Québec, G6V 3Z1 Canada; 2 Centre de recherche du CISSS Chaudière-Appalaches, Chaire de Recherche en Médecine D’urgence ULaval - CISSS Chaudière-Appalaches, Lévis, Canada; 3 Centre de Recherche sur les Soins et Services de Première Ligne, Université Laval, Québec, Canada; 4 Centre de Recherche Charles-Le Moyne-Saguenay-Lac-St-Jean-sur-les-Innovations-en-Santé, Longueuil, Canada; 5 Faculty of Medicine and Health Sciences, Université de Sherbrooke, Longueuil, Canada; 6 Institute of Public Health Research of the University of Montréal, Montréal, Canada; 7 Institut National D’excellence en Santé et Services Sociaux, Montréal, Canada; 8 Department of Social and Preventive Medicine, Université Laval, Québec, Canada; 9 Faculty of Medicine, Université Laval, Québec, Canada; 10 Department of Pediatric Emergency Medicine, CHU Sainte-Justine, Université de Montréal, Montréal, Canada; 11 Department of psychology, Université du Québec à Montréal, Montréal, Canada

**Keywords:** Emergency medicine, Rural, Case study, Stakeholders

## Abstract

**Background:**

Emergency departments (EDs) in rural and remote areas face challenges in delivering accessible, high quality and efficient services. The objective of this pilot study was to test the feasibility and relevance of the selected approach and to explore challenges and solutions to improve delivery of care in selected EDs.

**Methods:**

We conducted an exploratory multiple case study in two rural EDs in Québec, Canada. A survey filled out by the head nurse for each ED provided a descriptive statistical portrait. Semi-structured interviews were conducted with ED health professionals, decision-makers and citizens (*n* = 68) and analyzed inductively and thematically.

**Results:**

The two EDs differed with regards to number of annual visits, inter-facility transfers and wait time. Stakeholders stressed the influence of context on ED challenges and solutions, related to: 1) governance and management (e.g. lack of representation, poor efficiency, ill-adapted standards); 2) health services organization (e.g. limited access to primary healthcare and long-term care, challenges with transfers); 3) resources (e.g. lack of infrastructure, limited access to specialists, difficult staff recruitment/retention); 4) and professional practice (e.g. isolation, large scope, maintaining competencies with low case volumes, need for continuing education, teamwork and protocols). There was a general agreement between stakeholder groups.

**Conclusions:**

Our findings show the feasibility and relevance of mobilizing stakeholders to identify context-specific challenges and solutions. It confirms the importance of undertaking a larger study to improve the delivery of care in rural EDs.

## Background

Rural and remote emergency departments (EDs) in Canada provide an essential safety net in areas where access to alternative services (e.g. primary healthcare) is limited and where individuals have lower incomes, less education, less healthy lifestyles, higher mortality rates and shorter life expectancy compared to people living in urban areas [1–4]. Rural EDs face complex challenges in delivering accessible, quality and efficient services due to their long distance from referral centres, difficulties recruiting and retaining staff, limited access to specialists, and unequal presence of modern infrastructures [2, 5, 6]. These challenges require solutions tailored to rural and remote contexts. General standards of care and recommendations to improve delivery of care in EDs are often ill-adapted to rural contexts, in spite of repeated calls to this effect [7–10]. Moreover, several authors have warned against trying to implement “one size fits all” solutions to improve the delivery of care in rural and remote settings; instead, they suggest that local stakeholders should be engaged in identifying challenges and solutions adapted to context and needs [11–14]. Yet, this has never been done for rural EDs in the province of Québec, Canada.

In our study *Rural Emergency Care 360°* [15], we aimed to mobilize multiple stakeholders of rural EDs across Québec, to identify and implement context-specific challenges and solutions with the potential to improve the delivery of care in these settings. Before conducting our project in EDs across the province, the present pilot study aimed to:

1. Test the feasibility of mobilizing different stakeholder groups to identify challenges and solutions appropriate for improving rural EDs;
2. Explore specific challenges in rural EDs and potential solutions to improve delivery of care.

## Methods

### Setting

**Map 1:**
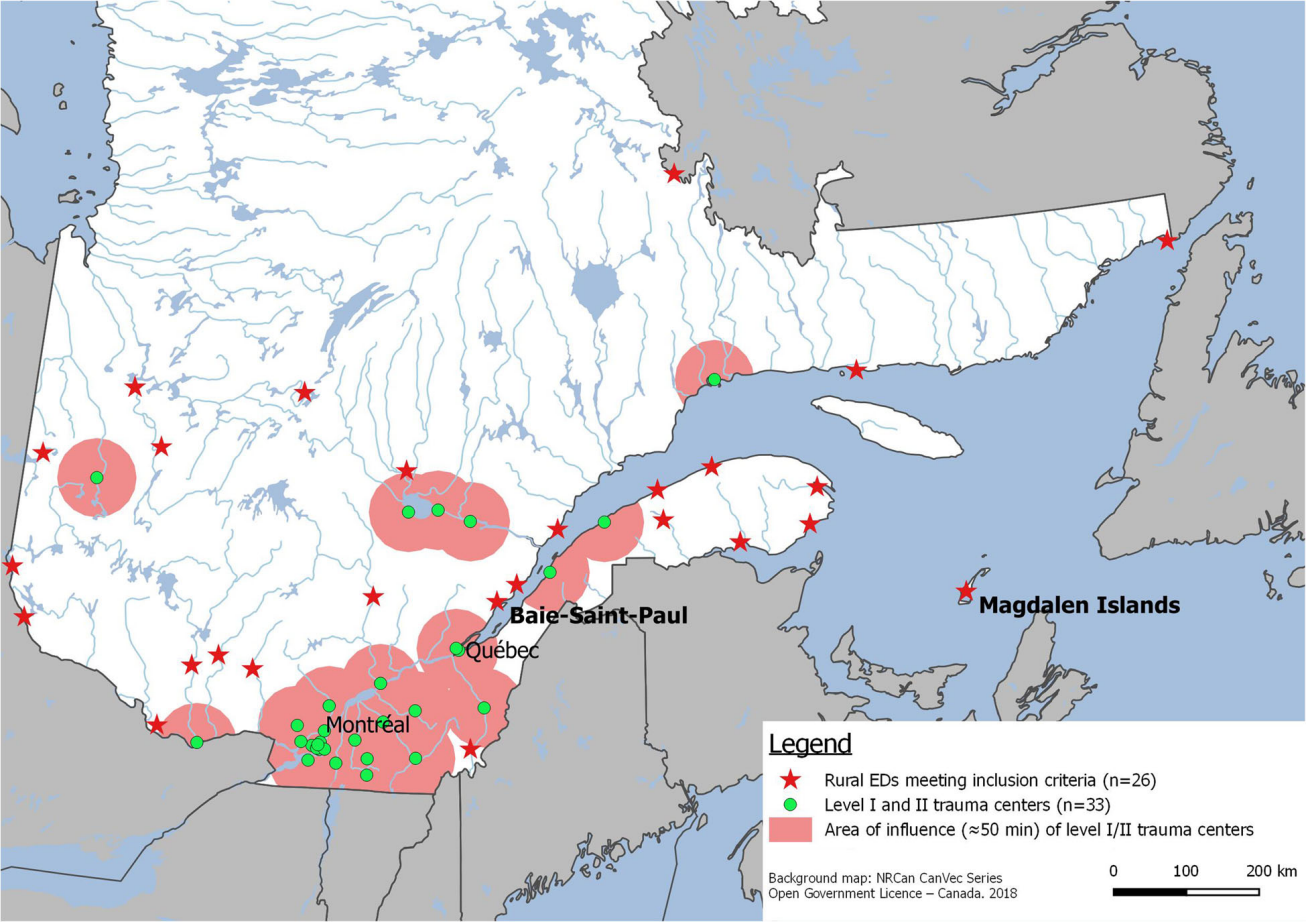
Location of Baie-Saint-Paul and the Magdalen Islands in the map of the 26 rural EDs that match our definition in the province of Quebec. Source: Our team. Background map: NRCan CanVec Series; Open Government Licence – Canada. 2018

This pilot study was conducted in Quebec, Canada, where 26 EDs meet our definition of a “rural ED”: located in a town of under 15,000 inhabitants [16], situated over 50 min driving time (Google Maps) from a secondary or tertiary trauma centre, and offering 24/7 medical coverage with hospital beds (Map 1). Since 2015, Quebec’s health and social services system consists of two governance levels: provincial (Ministry of Health and Social Services) and regional (Integrated Centers for Health and Social Services). The former is responsible for distributing resources, establishing policies and standards, coordinating and monitoring the health system; while the latter ensures accessibility, continuity and quality of general and specialized services (social, physical, hospital, long-term care, youth, rehabilitation) for the population in their region.

### Design & case selection

We conducted a multiple case study to explore challenges and solutions for improving rural EDs – an appropriate design for considering multiple stakeholders’ perspectives within their context [17].

The province of Quebec has 26 EDs that meet the definition of “rural” used in our previous work, which is based on the following criteria (1) located in cities with a population of less than 15,000 (2016 census data); (2) 24/7 physician coverage; (3) hospital with patient admission capability; and (4) located more than 50 min of ground transport from a level 1 or 2 trauma centre [18]. Of Québec’s 26 rural EDs, the research team selected, by convenience, two contrasting cases: Baie-Saint-Paul Hospital and Archipel Hospital at Cap-aux-Meules in the Magdalen Islands (see Map 1). Baie-St-Paul hospital was selected as it was located relatively close (92 km) to the research team and to a level I trauma centre. It had also successfully participated in our initial studies on rural emergency care [5, 19, 20]. Finally, the principal investigator had previously worked as a locum doctor in this ED and thus knowledge of local stakeholders was perceived as a facilitator in the context of this pilot study. The Magdalen Islands’ ED was selected because of its isolated location (islands in the St-Lawrence’s Gulf), that place it at the opposite of Baie-St-Paul on this particular aspect. The objective was to test our strategy and identify challenges and potential solutions in different contexts. The Magdalen Islands has nearly twice the volume of annual visits compared to Baie-Saint-Paul and has access to more medical specialities and a CT Scan. Baie-St-Paul’s inter-facility transfers are transported by ambulance, while Magdalen Island has to rely on an air ambulance system. All 26 rural EDs in the *Rural Emergency Care 360°* study will fall somewhere between one of these two “extremes” settings (close to an urban center vs. isolated), representing a diverse range of contexts. This case study selection strategy allows us to compare rural communities with each other, rather than with urban settings, uncovering disparities and similarities that will enrich our understanding of challenges and solutions [21].

### Data collection

The head nurses of the two hospitals received a previously tested quantitative survey [5] to provide a descriptive statistical portrait of each ED. The survey included, among others, questions on ED staff, access to diagnostic services, access to specialists, interfacility transfers, average wait times, volume and types of visits. Subsequently, an interview guide containing open ended questions was used to conduct semi-structured individual and group interviews with a range of stakeholders. Semi-structured interviews make it possible to identify the perceptions of interviewees on specific themes. A common interview guide was used and slightly adapted according to the type of interview (individual or group) and the type of participants. Interview questions related to 1) local context; 2) available health and social services; 3) participants’ perception of the ED’s situation and challenges; 4) existing or potential solutions to improve delivery of care in the ED and; 5) future considerations,. The selection of these five themes was based on literature and previous work [22]. The objective was to cover a fairly wide range of themes associated with rural health care.

In each ED, we selected a local “champion” to help identify participants and act as a knowledge broker. We recruited participants in each ED according to their position or engagement, diversity of profile (sex, age, profession, etc.) and interest in participating. Suggestions from the champion and snowballing were used to recruit additional participants until the members of the research team involved in preliminary analysis felt that further data collection was no longer adding to the analysis (data saturation) [23]. Participants were approached face-to-face by champions, and by phone and email by the research team. Participants were informed about the main researcher’s reasons for conducting the project and about the interviewer role. Group interviews were preferred with homogenous groups (citizens, nurses, prehospital staff, diagnostic resources). Individual interviews were conducted with stakeholders with a unique perspective (decision-makers, unique health professionals such as pharmacists, and elected representatives) or who were not available to participate in group interviews (all physicians and some nurses). An experienced research associate academically trained in qualitative research conducted (JPM, M.A. Anthropology, male) the interviews by telephone, videoconferences that took place at both hospitals or in person at the Baie-St-Paul hospital, from March to May, 2016. Socio-demographic data was collected on each participant. Interviews lasted one to 2 h, were audio recorded and transcribed. Only the interviewer and the participants were present during individual and group interviews. No repeat interviews were conducted.

In an attempt to foster local mobilization around emergency care, a conference on the study, organised in collaboration with artists and a local community group, was also presented in March 2018 in Baie-St-Paul.

### Analysis

Quantitative data was analysed using descriptive statistics (means and percentages), with Excel 16. Content analysis of qualitative data was conducted thematically and inductively [24] using NVivo11. Thematic coding was performed by MPR and CTP, research associates academically trained in qualitative research (parallel analysis of 40% of the interviews) and discussed to achieve consensus. Analysis and interpretation of qualitative data were achieved by discussions with the lead investigator (RF), co-investigators (JPF, HA, GD) and an expert in qualitative research/rural health (HS). Preliminary analysis were discussed during two focus groups (one in each ED) with diverse stakeholders (nurses, doctor, manager, champion). Quotes presented below were translated from French to English by the research team and validated by a professional medical translator (LB).

## Results

### Participants

A total of 33 semi-structured individual interviews and nine group interviews were conducted with 68 participants of diverse backgrounds (see Table 1).

**Table 1:** Participants in qualitative interviews

Types of participants	Number of participants		
	Magdalen Islands	Baie-St-Paul	Total
COMMUNITY ➔ concerned citizens ➔ elected officials ➔ patients / caregivers	8	7	15
PHYSICIANS ➔ GPs / specialists	6	7	13
NURSES AND DECISION-MAKERS* ➔ nurses ➔ managers ➔ health system decision-makers *Presented together to ensure confidentiality	6	4	10
EMERGENCY MEDICAL SYSTEM (EMS) ➔ first responders ➔ paramedics ➔ EMS decision-makers	4	10	14
OTHER HEALTH PROFESSIONALS ➔ psychosocial ➔ diagnostic services ➔ pharmacy	10	6	16
Total	34	34	68

### ED descriptive statistical portraits

Table 2 presents a brief statistical portrait of the EDs.

**Table 2:** Portrait of emergency services at Baie-St-Paul and the Magdalen Islands – selected statistics

Variables measured (2014–15)	Magdalen Islands	Baie-Saint-Paul
Number of annual visits	21,284	12,940
Triage		
Level 1–3	8.3%	11.0%
Level 4–5	91.7%	89.1%
Number of stretchers	6	6
Wait time to see a doctor	120 min	66 min
Average time in emergency (hours)	11.3 h	9.5 h
Distance to trauma center level 1^a^	1157 km (including ferry)	92 km
Number physicians		
Full time	4	7
Part-time	6	3
Number of emergency care nurses		
Day	3	5
Evening	3	3
Night	2	2
Receptionist	Yes	No
Porter	No	Yes
Social worker	Yes	Yes
Liaison nurse	Yes	Yes
Laboratory	Yes	Yes
Radiology	Yes	Yes
Intensive care unit	Yes	No^b^
CT scan	Yes	No
Surgeon	Yes	Yes
Respiratory therapist	Yes	Yes
Anesthetist	Yes	Yes
Psychiatrist	Yes	Yes
Internist	Yes	No
Obstetrician/gynecologist	Yes	No
Orthopedist	No	No
Pediatrician	No	No
Pharmacist	Yes	Yes
Number of interfacility transfers annually	204	488

^a^Distances obtained using Google Maps

^b^Baie-Saint-Paul does however have an intermediary care unit

### ED contexts

Stakeholders described the influence of rural context on challenges faced by the EDs, particularly with regards to proximity/isolation, population and strengths of the community. On one hand, participants in both EDs highlighted that the attractiveness of their region (e.g. landscapes, charm) could be an asset in recruiting and retaining health professionals. On the other hand, they noted that geographic distance and isolation could be a barrier to recruitment as well as a challenge for patients’ medical transportation, especially during bad weather which is relatively frequent in winter. This barrier seemed more prominent in the Magdalen Islands, where the smaller number of interfacility transfers (204 annually vs. 488 in Baie-St-Paul; see Table 2) was attributed in part to the complexity brought by the geographic isolation of the islands, compared to Baie-Saint-Paul:

*What I feel is that in the [Magdalen Islands], they are further away. So, they're really going to try harder to keep their patients, to do the operations.... While here [in Baie-Saint-Paul], we have easy access to specialists [in Quebec], faster, and it's closer too.* (Baie-St-Paul, health professionals, group interview 5)

While the presence of an ICU and a CT Scan in the Magdalen Islands does allow this ED to treat more cases locally than Baie-Saint-Paul’s ED, this health professionals also point to the unformal reasons underlying transfers for some more ambiguous cases.

In both the Magdalen Islands and Baie-Saint-Paul, stakeholders discussed pressure on the ED related to the aging population, namely due to the exodus of young people, the aging of the remaining population and the influx of retirees in their regions. Both EDs also faced specific challenges due to tourism-related seasonal fluctuations in population. This seasonal influx of population was said to increase the number ED visits during the summer months and to pose a challenge for resource allocation and for monitoring performance indicators.

In addition, social proximity – in these regions where “everyone knows everyone” – was identified as a distinctive feature of rural EDs as it poses a challenge to patient confidentiality and influences how patients use health services (e.g. visit based on the staff on-duty). Conversely, community members and some care givers stressed that this social proximity was linked to patients having more social support and stronger social capital in the community, coupled with more humane care in the ED.

Stakeholders in the two EDs also emphasized the strong historical and cultural attachment of communities to their healthcare, and the importance of a strong ED in a context of remoteness (in case of emergencies) and demographic decline (for attractiveness of the region):

*It goes further than that. There’s a presence here that is special … The ties people have with the healthcare system, here, it’s almost an emotional tie, more than just a relationship of client and service*. (Baie-St-Paul, citizen, individual interview 14)

Furthermore, while stakeholders recognized the challenges of delivering accessible, high quality and efficient ED care in their setting, many also expressed pride in how creative and flexible rural EDs could be in implementing innovative solutions to overcome these challenges:

*You always think that innovation happens only in tertiary hospitals, because that’s where all the cutting-edge research happens and everything. But in fact, it’s precisely because there’s less bureaucracy here and people are less dogmatic, there are many things that we do here that I’d say are more innovative than what I’ve seen in the city. (Magdalen Islands, physician, individual interview 24)*

### Challenges and solutions in rural EDs

**Fig. 1:**
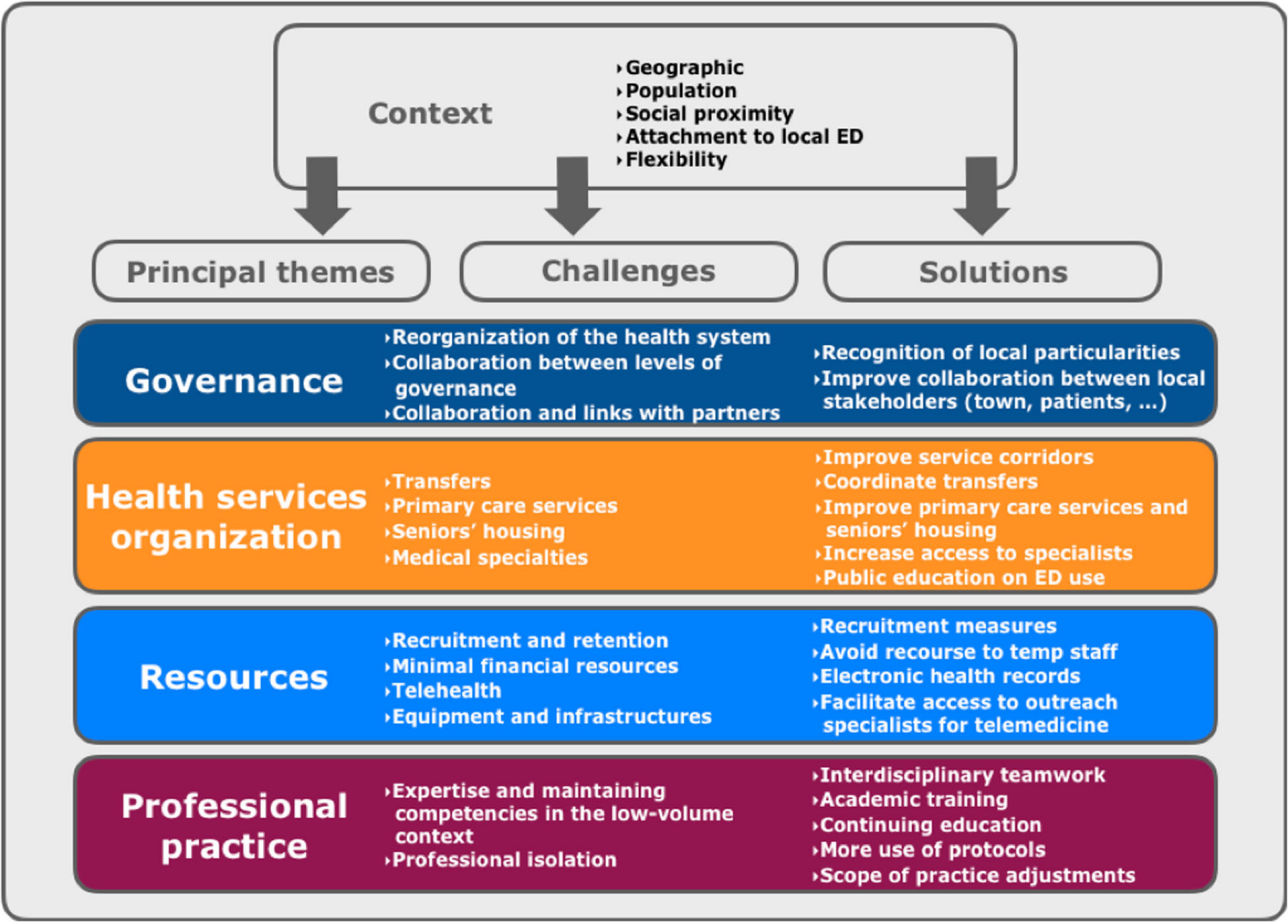
Qualitative data summary

Challenges and solutions specific to the two rural EDs were related to: 1) governance and management; 2) health services organization; 3) resources; and 4) professional practice. Figure 1 summarizes emerging themes, which are described in more detail below.

#### Governance & management

In both the Magdalen Islands and Baie-Saint-Paul, all stakeholders groups emphasized the challenge of managing the ED efficiently in a small rural setting, while juggling volumes of visits, costs, safety, staffing and performance:

*To have enough staff to be able to operate on the three shifts, even though we know that at night it is much quieter in an emergency room like [here]. But we still have to keep the teams in place on the three shifts. So it has an impact on our statistical performance. Then it has an impact on costs too.* (Magdalen Islands, citizen, individual interview 21)

In terms of governance, Baie-Saint-Paul had recently loss administrative autonomy as a result of recent province-wide mergers of local health centers into regional centers – mergers that had spared the Magdalen Islands. While the impacts on the ED were still unknown at the time of interviews, stakeholders in Baie-Saint-Paul were worried that their “local colour” would be lost in the merger: they feared local adaptations to context-specific challenges would be discarded and quality of care in the ED would suffer. To address this challenge, stakeholders discussed the importance of establishing governance structures with adequate local representation of rural contexts. They also recommended improved collaboration between local stakeholders, including the municipality, the police and community organisations.

Stakeholders stressed that current standards of care, like the provincial *Emergency Management Guidelines* [25], were ill-adapted to rural EDs and that new standards of care should be flexible to their local particularities:

*For sure the* [provincial] *Emergency Management Guidelines are a help, for us, but they’re not the be-all and end-all. And we can’t always follow everything in it. I don’t know a single emergency department in a small region which follows them all. We try to follow them as closely as we can, but it’s impossible. And often, we find that our situation isn’t taken into consideration in the guidelines. They’re based on what happens in big hospitals. (Magdalen Islands, nurses and decision-makers, individual interview 8)*

#### Health services organization

In both regions, stakeholders pointed to limited availability of local primary healthcare and long-term care services, as a driver of the number of ED visits and wait time in EDs. Participants in both sites also stressed the lack of local access to psychosocial services and the pressure it caused on rural EDs. They explained that, in the absence of appropriate alternatives, patients relied more heavily on rural EDs to meet their needs:

*Currently, like intermediate resources and private residences for seniors, we are severely lacking those. We have long … we’ve exceeded the six-month wait for a private residence [ … ] Because they have to wait, and wait, and wait, families are exhausted. Which means that those people end up at the emergency department and are hospitalized. (Baie-St-Paul, nurses and decision-makers, group interview 4)*

The need to increase the availability of primary healthcare and long-term care in these rural areas was a recurring topic among all stakeholder groups. Some stakeholders also talked about educating rural populations (pamphlets, interventions in the ED, etc.) about appropriate ED use and existing services.

Challenges related to interfacility transfers were a salient and recurring theme. As presented in Table 2, transfers represent 0,96% of all visits in Magdalen Islands and 3,77% in Baie-St-Paul. For patients and their families, costs incurred and distance from their communities could be difficult, particularly for the elderly. For health professionals, organizing medical transportation and transferring medical responsibility to the receiving facility was described as time consuming. Having to justify and defend the clinical decision of transferring a patient was also said to be challenging for rural ED physicians. For paramedics, transfers involved wait time for transportation (e.g. plane) or travelling long distances, sometimes in difficult weather conditions. Transferring patients could also monopolize ambulances, leaving fewer available to answer emergency calls in the region. Improving coordination, finding alternative modes of transportation (e.g. snowmobiles, helicopters) and establishing interfacility service agreements for transfers were solutions suggested by stakeholders in both EDs.

#### Resources

Participants identified lack of resources to deliver quality care in rural EDs as a major challenge. Financial and human resources were often seen as insufficient and poorly adapted. Recruiting and retaining ED staff was seen as one of the biggest obstacles to providing quality care in EDs. As we can see in Table 2, a small team of 10 physicians shared the 24/7 coverage in each of the two EDs, leaving little room for the unexpected (health problem, maternity leave, etc.). The EDs sometimes had to rely on temporary staff and locum to avoid gaps in services, but this was viewed as less than ideal for efficiency, continuity and quality of care. Stakeholders identified a need for adapted recruitment measures that would make working in rural EDs more appealing, including exposing health professionals to rural EDs during their training, promoting the advantages of working in rural EDs (e.g. generally less hectic environment than in urban EDs, expanded scope of practice, close collaboration with a small team, attractiveness of the region, quality of life), and appropriate financial incentives. Encouraging a broader use of electronic medical records was mentioned as a solution to help with continuity of information in the context of high rates of staff turnover.

Accessing an adequate range of specialists and diagnostic equipment was also said to be difficult in both EDs. While citizens expressed that it would be desirable to broaden the range of specialities available in their hospital, health professionals stated that the priority should be to achieve basic coverage of essential specialities like anaesthetists, surgeons and radiologists. Many physicians also noted the role they felt they had to play in advocating for sufficient resources in their hospital.

Stakeholders pointed to partnerships with community organisations, interfacility agreements for access to diagnostic equipment and travelling specialists as promising solutions to increase resources available in rural EDs. Telehealth and other eHealth innovations were often cited as interesting solutions to increase access to specialists; but many barriers remained before they could fully be implemented, namely addressing technological considerations (e.g. limited bandwidth in rural areas), and ensuring coverage by distant specialists.

#### Professional practice

Health professionals acknowledged the challenge of having a larger scope of practice in rural and remote EDs, because of their limited access to different specialities. Participants linked this to a need to adjust provincially-defined professional scopes of practice to rural contexts. Conversely, the difficulty of maintaining a large scope of expertise and competencies in the context of low volumes was also mentioned.

To address these challenges, participants suggested an increase in the use of care protocols. Moreover, interdisciplinary teamwork appeared to be the main solution applied in both regions. Continuing education was also pointed to as a means to improve professional competencies, but many participants underlined the importance of offering opportunities for hands-on practice during formal courses or dedicated training periods.

## Discussion

During this pilot study, we found that engaging a range of local stakeholders in these types of discussions: 1) is a feasible approach, and 2) can help explore context-specific challenges and identify relevant solutions with the potential to improve delivery of care in different rural EDs.

### Feasibility of our approach

This pilot study gave us an opportunity to test the feasibility of our approach. Conducting an exploratory multiple case study, which combined a quantitative survey and qualitative interviews with stakeholders, allowed us to get an overview of each ED’s characteristics as well as a rich understanding of context, specific challenges and potential solutions. While we anticipated that different stakeholder groups might have opposing views, our data shows that challenges and solutions were generally agreed upon by the different types of stakeholders. This is consistent with another study conducted in Québec that found general agreement between health professionals and decision-makers on solutions to improve the health system [26]. Our study adds to this by mobilizing local citizens as well. Our approach provided us with very rich data, as different stakeholder groups provided complementary views and information, which allowed us to gain a more in depth understanding of each ED.

Our data collection tools were effective for achieving our objectives. However, we will shorten the interview guides for the next phase of *Rural Emergency Care 360°* project to focus on high-priority data (e.g. barriers and facilitators of potential solutions). Because of its rigour and relevance, our coding grid will serve as a strong basis in our study across the province.

### Context-specific challenges and solutions of rural EDs

Our findings suggest that local contexts of rural and remote settings (e.g. geographic isolation, demographic concerns, social proximity, attachment to the ED, flexibility) considerably affect the delivery of care in rural EDs and the potential solutions to improve it. The data also points to the importance of the global context in with rural EDs are evolving (e.g. financial constraint, health policies, regional development) in understanding the barriers and facilitators that may influence implementation of solutions.

Despite differences in their contexts, stakeholders in Baie-Saint-Paul and the Magdalen Islands discussed similar challenges and solutions related to governance and management, resources, health services organization and professional practice. This suggests that despite considerable differences between them, fostering exchanges between rural EDs to share potential solutions is relevant. While these solutions may require some context-specific adaptations, it seems that focussing on exchanges between rural EDs could help address shared concerns in a more relevant fashion that would be possible in a study on EDs in general.

Furthermore, the themes emerging from our data for rural EDs align with challenges in delivering healthcare in rural settings described in the literature: difficulties recruiting and retaining staff, limited access to modern equipment and technologies, challenges with centralized governance, large scope of practice, lack of resources, limited access to comprehensive health services and poorly adapted standards of care [2, 5, 6, 11, 27]. While our findings provide some context-specific nuances, many of the solutions identified in the two rural EDs are in keeping with published recommendations and guidelines, namely telehealth, continuing education, interdisciplinary teamwork, interfacility agreements, recruitment strategies and adequate transportation [10, 25, 28].

In addition to identifying challenges and solutions, without prompt, stakeholders repeatedly highlighted the strengths of rural EDs. They strive to deliver accessible, high quality and efficient care despite limited means – creatively and flexibly adapting to their contexts to overcome challenges. Stakeholders also spoke with pride about the strong social capital and close ties with community. These findings point to important facilitators of change which can be harnessed to improve the delivery of care in rural EDs.

### Mobilizing stakeholders to foster implementation of solutions in rural EDs

Our findings highlight that many barriers remain to improving the delivery of care in rural EDs. While current *Emergency Department Management Guidelines* in Québec include a small section on rural EDs [25], it is clear from our findings and from previous work [5, 29] that the use of these guidelines is limited in rural EDs. Mobilizing rural stakeholders in a dialogue about challenges and solutions may be a promising approach to foster improvements. Our approach can help produce recommendations that are both evidence-based and better adapted to real-world constraints. Furthermore, mobilizing multiple stakeholder groups in at the stage of a pilot study to discuss challenges and potential solutions can help engage them in later adopting and implementing solutions identified in the context of our large-scale project. Engaging local stakeholders is often overlooked in trying to move from evidence into implementing change in practice [30, 31]. Engaging with local decision-makers, a range of health professionals and citizens can help increase likelihood that other individuals from these groups will also support the implementation of identified solutions in later stages, improve readiness for change, reduce resistance and increase the acceptability and relevance of solutions to local contexts [31–34].

### Strengths and limitations

Combining quantitative and qualitative data provided us with a more comprehensive understanding of the challenges and solutions in the two rural EDs. The quantitative data we needed for this project was not readily available in public reports, in which statistics are amalgamated by regional organization and not differentiated for each ED. Surveys were thus filled out by a single representative from each ED, with limited validation. Furthermore, because the pilot study focuses on only two EDs, the scope of the quantitative analysis we could conduct was limited. The *Rural Emergencies Care 360°* project will allow for a more in-depth analysis of quantitative data.

For the qualitative data, we used different strategies to increase quality and rigor [24]: conducting group interviews only in homogeneous groups to allow all voices to be heard, conducting inductive analysis to stay close to participants’ statements, coding by two research associates and consultation with experts in qualitative research and rural EDs. We found that the use of champions was particularly effective to recruit participants and maintain ties in each setting. However, this may also have created a bias in the recruitment process. Because the number of participants and type of interview varied, certain stakeholder groups may have had more influence on our findings. However, we made a deliberate effort to ensure fair representation of the views of each group. The citizens selected for interviews were highly informed and may not be representative of the whole population. Our exploratory pilot study was conducted in only two rural EDs in Québec, which may limit transferability of our findings. Nonetheless, we chose two EDs in different contexts, which we described in detail, to improve transferability. The scope of our data in this exploratory pilot study therefore offers a solid foundation for future work. We referred to the COREQ checklist to ensure rigorous reporting on this pilot study [35].

## Conclusions

This pilot project, in which the process is as important as the conclusions, lays the cornerstone for a larger project that goes well beyond a qualitative approach and statistical portrait of rural EDs. Our *Rural Emergency Care 360*° [15] study aims to support the rural emergency community to take ownership of results. To this end, a panel of experts will be formed to transform solutions identified by rural EDs in our study into useful contextually-adapted recommendations. Discussions are also underway to develop a living-laboratory in the Baie-St-Paul ED to test and evaluate solutions to improve rural EDs: our pilot project has indeed been effective in mobilizing a diverse set of stakeholders.

## Data Availability

Research data cannot be shared publicly because individual privacy could be compromised. The detailed research report provides more information data collected. It is available in French upon request.

## Abbreviations

COREQ: Consolidated criteria for reporting qualitative research
ED: Emergency department
EMS: Emergency medical service
GP: General practitioner
